# Proposal for a definition of "Oligometastatic disease in pancreatic cancer"

**DOI:** 10.1186/s12885-019-6448-9

**Published:** 2019-12-30

**Authors:** Alexander I. Damanakis, Luisa Ostertag, Dirk Waldschmidt, Fabian Kütting, Alexander Quaas, Patrick Plum, Christiane J. Bruns, Florian Gebauer, Felix Popp

**Affiliations:** 1 Department of General, Visceral, Cancer and Transplantation Surgery, University Hospital of Cologne, Cologne, Germany; 2 Department of Gastroenterology, University Hospital of Cologne, Cologne, Germany; 3 Institute of Pathology, University Hospital of Cologne, Cologne, Germany

**Keywords:** Pancreatic adenocarcinoma, Metastasis, Chemotherapy, Surgery, Oligometastasis

## Abstract

**Background:**

To date, patients with metastasized pancreatic ductal adenocarcinoma (PDAC M1) are regarded as a uniform collective. We hypothesize the existence of oligometastatic disease (OMD): a state of PDAC M1 disease with better tumor biology, limited metastasis, and increased survival.

**Methods:**

Data of 128 PDAC M1 patients treated at the University of Cologne between 2008 and 2018 was reviewed. Interdependence between clinical parameter was calculated using the Mann-Whitney U-Test. Survival curves were generated using the Kaplan-Meier method and analyzed using the log-rank test.

**Results:**

Eighty-one (63%) patients had metastases confined to one organ (single organ metastasis, SOG) whereas the remaining 47 (37%) showed multiple metastatic sites (multi-organ metastasis, MOG). Survival analysis revealed a median overall survival (OS) of 12.2 months for SOG vs 4.5 months for MOG (95% CI 5.7–9.8; *p* < 0.001). We defined limited disease by the presence of ≤4 metastases in liver or lung. Limited disease together with CA 19–9 baseline < 1000 U/ml and response or stable disease after first-line chemotherapy defined OMD. We identified 8 patients with hepatic metastases and 2 with pulmonary metastases matching all OMD criteria. This group of 10 (7.8%) had a median overall survival of 19.4 vs 7.2 months compared to the remaining patients (95% CI 5.7–9.8; *p* = 0.009).

**Conclusion:**

We propose a definition of oligometastatic disease in PDAC including anatomical criteria and biological criteria reflecting better tumor biology. The 10 OMD patients (7.8%) survived significantly longer and might even benefit from surgical resection in the future.

## Background

Pancreatic ductal adenocarcinoma (PDAC) is still one of the most challenging cancers to treat for surgeons, medical and radiation oncologists alike. It has a dismal prognosis, with a median 5-year survival rate of 3%, and is the fourth leading cause of cancer-related deaths worldwide [1, 2]. One major challenge for the surgical treatment of PDAC is that only approximately 15% of patients are resectable upon diagnosis, whereas 40% show distant metastasis and 45% present with locally advanced unresectable disease. The median survival after surgical R0 resection is 22 months [3]. Compared to gemcitabine monotherapy, Conroy and colleagues [4] showed a significant increase in median overall survival of 54.4 months applying adjuvant FOLFIRINOX in resectable non-metastasized PDAC in a selected group of patients.

Although progress has been made in the multimodal treatment of patients with resectable and locally advanced PDAC, one principle of therapy still prevails: surgical resection of the primary tumor and metastases in PDAC M1 patients, regardless of individual tumor and patient characteristics, is explicitly not recommended in clinical guidelines [5, 6]. PDAC M1 patients are referred to palliative chemotherapy or best supportive care independent of the number and localization of metastases or other individual patient characteristics. In this situation, the most effective palliative chemotherapeutic regimens can improve median overall survival from 6.7 to 8.5 months (gemcitabine plus nab-paclitaxel) and 6.8 to 11.1 months (FOLFIRINOX), respectively [7, 8].

Considering the abovementioned modern combination chemotherapies and safer pancreatic surgery, a new approach to metastatic disease in PDAC comes into focus in current research [9, 10]. Retrospective data suggest that resection of the primary tumor and solitary liver metastases can improve overall survival, leading to reported median survival rates of 14.4 and 12.2 months, respectively. In addition, only a minority of patients received neoadjuvant treatment in these collectives [11, 12]. These encouraging results suggest that a well selected subset of PDAC M1 patients could benefit from multimodal therapy, including surgery to further prolong overall survival. Of course, all PDAC M1 patients receive chemotherapy first. This means that patients who are eligible for surgery have to meet the following requirements: The tumor and the metastases are resectable, there is no progress under chemotherapy and they will presumably benefit from the operation. The last point is difficult to anticipate. Those benefiting from the operation would consist of patients with “better” tumor biology, but how should this “better” tumor biology be defined? We believe that these are the patients who live significantly longer under standard therapy. Clinical experience shows that there are indeed patients who survive significantly longer. Thus, we hypothesize the existence of “oligometastatic disease” (OMD) in pancreatic cancer that identifies patients with better tumor biology. We further hypothesize that OMD patients benefit from surgery in the sense that they live substantially longer.

Our aim is to identify clinical criteria reflecting “better” tumor biology in PDAC M1 patients. We think that patients with metastatic disease do not represent a uniform group. Patients with only a few metastases restricted to a single organ are defined here as “*limited disease*.” This definition relies solely on anatomical means. Even though the presence of *limited disease* is a prerequisite for the resectability of metastatic pancreatic cancer, it is a different condition than OMD. We believe that patients with OMD are the ones qualifying for surgical resection within a multimodal treatment approach.

## Methods

Data of metastatic pancreatic cancer patients treated at the University of Cologne between 2008 and 2018 was collected retrospectively and maintained using an Excel-based database. To that end, patients were identified using ICD 10-based queries (C25.1–3) in the clinical information system. All available patient data was reviewed thoroughly by one experienced surgeon. No patient included in our study underwent any form of tumor resection, neither before chemotherapy treatment, nor over the course of palliative treatment.

We retrospectively assessed clinical data, including, sex, age at diagnosis and all chemotherapy regimens the patient received during treatment of the disease and entered them into the database. Patients were only included in the analysis when contrast-enhanced, multiple detector computed tomography (CT) of the abdomen and thorax or MRI imaging was available. The time of PDAC M1 diagnosis was defined as the first CT or MRI scan revealing tumor and distant metastasis. Presence of pancreatic ductal adenocarcinoma had to be proven histologically, either via biopsy of one of the metastases or the primary tumor. The diagnosis of distant metastasis had to have been made at the time of initial diagnosis before the beginning of chemotherapy treatment. Follow-up information was obtained from the institution’s outpatient clinics, our clinical information system or the offices of the appropriate general practitioners. When the date of death was not recorded, patients were included but censored at the last recorded contact.

In summary, the following criteria had to be met in order for a patient to be included in the analysis:

- No surgery for primary tumor or metastases at any time during treatment.
- CT and/or MRI image available and sufficient to identify the primary tumor as well as distant metastases. The date of this radiological examination was defined as the timepoint of PDAC M1 diagnosis.
- Laboratory parameters of CRP, LDH, Bilirubin, CA 19–9 and CEA had to be available at the time of diagnosis.
- PDAC had to be histologically proven (via biopsy of metastases or the primary tumor).
- Therapeutic regimens the patient received during treatment of the disease had to be known.
- Time of death or last follow-up date available.
- As cholestasis can influence the CA 19–9 value, we included baseline CA 19–9 when a patient had a normalized bilirubin (< 1.2 mg/dl) after stenting.

This retrospective study was performed according to the criteria of the “Ethics Commission of Cologne University’s Faculty of Medicine”. According to the Ethics Vote, the collection of consent forms was not required.

### Statistical analysis

For statistical analysis we utilized IBM SPSS Statistics for Mac (Version 21; IBM Corp, Armonk, NY). As this is a retrospective study, some clinical information was missing. Whenever that was the case, we calculated relative percentages.

As described previously [11], we assessed associations between categorical variables with the χ^2^ and Fisher exact test displayed in cross tables. Differences in nonparametric groups were calculated by the Mann-Whitney U test. We used the Kaplan-Meier method to estimate the probability of the death event. In case when there was no death event recorded, patients were consecutively censored at the date of the last contact. The Log-rank tests and the exact stratified log-rank tests were used to compare survival. We applied univariate and multivariate analyses for prognostic factors using the Cox regression model. All tests were 2-sided.

## Results

Retrospective queries identified 566 patients who were treated because of metastasized pancreatic tumors from 2008 to 2018. Of the 566 initially identified patients, 128 patients with histologically confirmed PDAC M1, sufficient documentation of the clinical course and radiological imaging met the inclusion criteria. Of those 128 patients, 43 participated in clinical trials (ACCEPT [NCT01728818] *n* = 12, AFFECT *n* = 2, RESOLVE (PCYC-1137-CA) *n* = 7 [13], RASH (ML22774) [NCT01729481] *n* = 1, KÜPTAC NIS (ML23024) *n* = 1 [14], JANUS-1 *n* = 2 [15]).

### Patient data

Altogether, 128 patients (median age 66 years, range 53–89 years) meeting the inclusion criteria were referred to palliative chemotherapy due to stage IV disease. Analysis revealed that 111 received chemotherapy treatment, whereas 17 either could not receive therapy due to acute medical conditions or refused therapy. Ten patients were still alive at the close of analysis.

### Characterization of primary tumor and extrapancreatic tumor spread

**Fig. 1:**
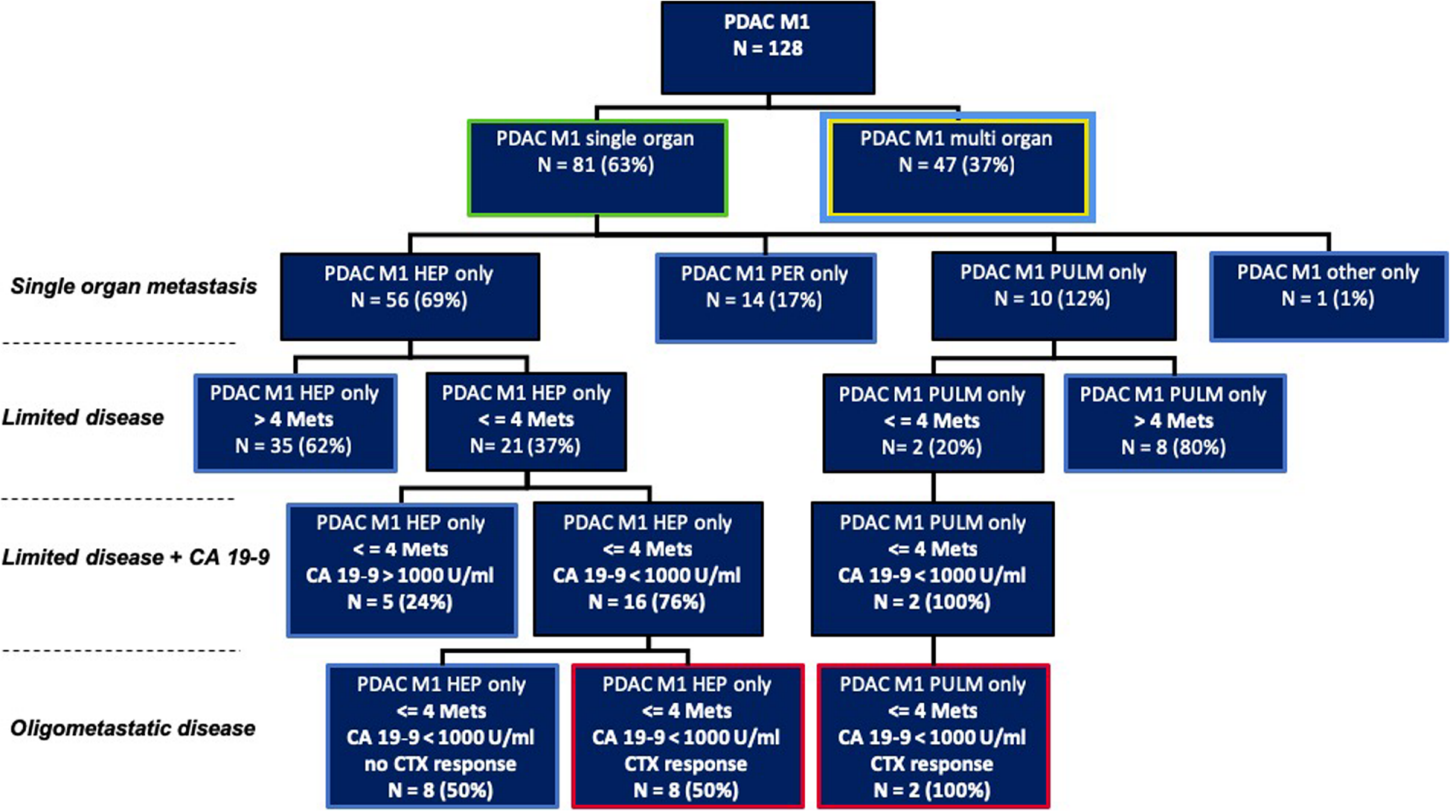
Metastatic pattern of single organ metastasized patients and application of OMD criteria

Following our abovementioned criteria, 128 PDAC M1 patients could be included in the analysis. Eight-one (63%) patients had metastatic spread in a single organ and 47 (37%) had a multi-organ manifestation upon diagnosis (Fig. 1).

### Single organ metastasis group (SOG)

The majority of single organ metastases were located in the liver (*n* = 56 of 81 patients, 69%) (Fig. 1). Fourteen (17%) patients in the single-organ group had peritoneal metastatic spread only, 10 patients (12%) showed pulmonary metastasis only. In order to more precisely define “*limited disease*,” we split the group according to the number of metastases in patients having up to 4 liver/lung metastases and 5 or more liver/lung metastases (Fig. 1). Twenty-one (37%) of the 56 patients had 4 or fewer intrahepatic lesions, while only 2 (20%) of the 10 patients with lung metastases had 4 or fewer intrapulmonary lesions. Altogether, 23 (41%) of the 56 SOG patients had anatomically *limited disease*. Apart from this morphological approach, in a second step we further stratified our *limited disease* group according to a CA 19–9 baseline threshold of 1000 U/mL (with bilirubin at the same time below 2 mg/dL). Both patients with pulmonary *limited disease* had a CA 19–9 value below 1000 U/mL upon diagnosis, whereas 16 (76%) of the 21 with hepatic *limited disease* fulfilled this criterion (Fig. 1). As will be further elucidated, we consider low CA 19–9 (< 1000 U/mL) as a marker of better biology. In order to further distinguish OMD from *limited disease* we added another clinical parameter in which we classified those patients by their response to first-line chemotherapy. We considered patients with single organ metastasis, fewer than 5 metastases in the liver or the lung, a CA 19–9 baseline value below 1000 U/mL (with bilirubin < 2 mg/dL) and responsive or stable disease after initial chemotherapy as having OMD. Responsive disease and stable disease were assessed using RECIST criteria. In the liver-only group, 8 (14%) of 56 fulfilled all the previously mentioned criteria, while 2 (20%) of 10 in the lung-only group fulfilled all criteria (Fig. 1). Those patients are described in more detail in Table 2.

### Multi-organ metastasis group

**Fig. 2:**
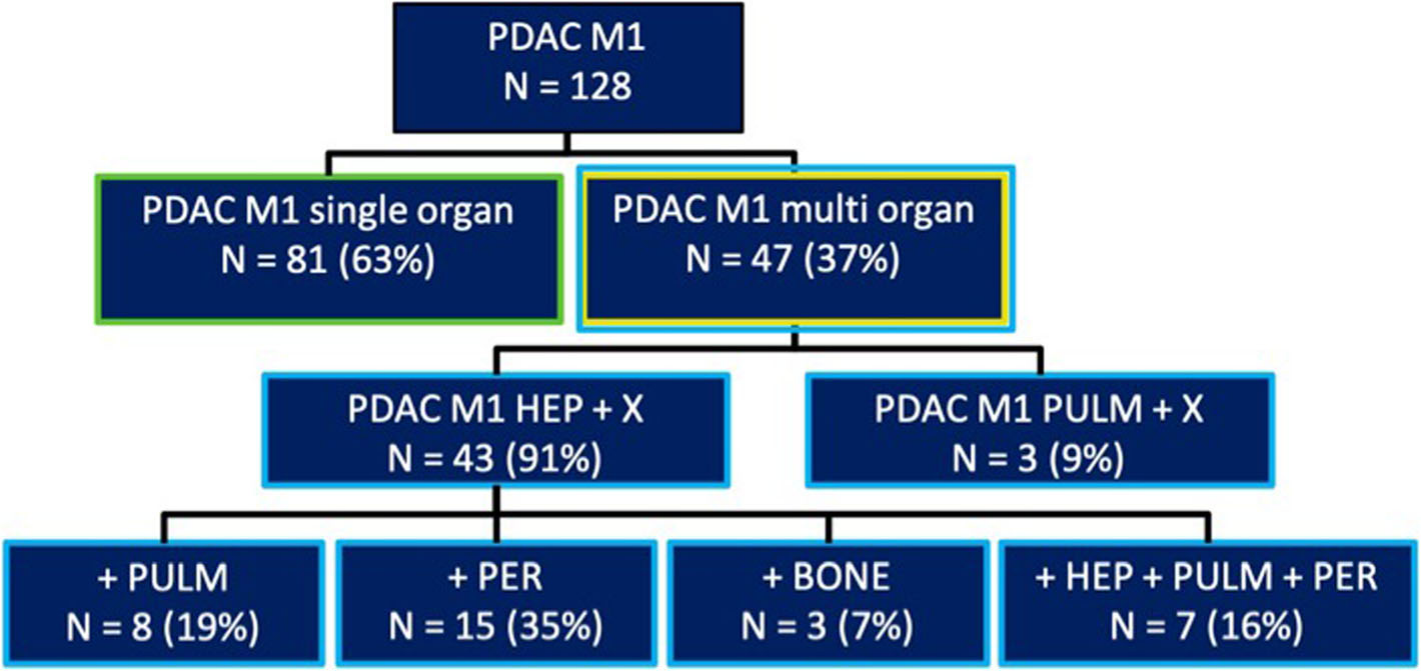
Metastatic pattern of multi-organ metastasized patients

We observed hepatic metastasis in 43 of 47 cases (91%, Fig. 2) in patients with multi-organ metastasis (Fig. 2). Of the 4 patients without liver metastases, 3 had pulmonary metastases. Additional pulmonary metastases were present in 8 patients (19%). Eleven patients (25%) of the 43 with hepatic metastases had only up to 4 metastatic lesions, whereas the remaining 39 patients had diffuse hepatic metastases.

### Chemotherapy

All patients were intended to receive palliative chemotherapy in our multidisciplinary tumor board. Of the 128 patients, 17 (13%) did not receive chemotherapy due to rapid deterioration of their health status or patient refusal. Ninety-eight (88%) patients received first line Gemcitabine-based chemotherapy, with the majority receiving Gemcitabine monotherapy (*n* = 40, 36%), followed by Gemcitabine with nab-Paclitaxel (*n* = 21; 19%) and Gemcitabine with Erlotinib (*n* = 15; 14%). The more aggressive FOLFIRINOX regimen was administered in 4 cases (4%) as a first line therapy. Of the 10 patients matching our criteria defining OMD, 9 received Gemcitabine-based regimens (90%), and 1 patient received Capecitabine (10%) (Table 1). Three patients (30%) received Gemcitabine monotherapy as first line treatment, 2 patients (20%) received Gemcitabine with Erlotinib, 2 patients (20%) received Gemcitabine with nab-Paclitaxel, 1 patient (10%) received Gemcitabine in combination with Afatinib and 1 patient (10%) received Ibrutinib or placebo and nab-Paclitaxel with Gemcitabine). Of the 103 patients that did not match our criteria for OMD who received chemotherapy, the majority (89%; *n* = 92 patients) received a Gemcitabine-based treatment as well.

**Table 1:** Patient data, first line chemotherapy and laboratory results of the whole patient collective, grouped into the patients matching our oligometastatic disease (OMD) criteria and those who do not

	Oligometastatic disease (OMD) *n* = 10	No oligometastatic disease (noOMD) *n* = 118	Mann-Whitney U Test (*p*-value)
Sex			
Male (%)	7 (70)	73 (61)	
Female (%)	3 (30)	47 (39)	
Age upon diagnosis, median (minimum; maximum)	65 (53;89)	66 (30;84)	0.945
Primary tumor: size [cm^2^], median (minimum; maximum)	14.5 (4.4;21.6)	12.0 (0.8;68.4)	0.758
Primary tumor: max. diameter [cm], median (minimum; maximum)	4.1 (2.4;9.0)	4.0 (1.0;10.4)	0.348
Primary tumor: Location			
Head, n (%)	2 (20.0)	53 (46.5)	
Body, n (%)	4 (40.0)	17 (15.8)	
Tail, n (%)	4 (40.0)	42 (37.7)	
Chemo: First line			
None, n (%)	0 (0)	17 (14.2)	
Gemcitabine, n (%)	3 (30.0)	38 (31.7)	
Gemcitabine + Erlotinib, n (%)	2 (20.0)	13 (10.8)	
Gemcitabine + nab-Paclitaxel, n (%)	2 (20.0)	19 (15.8)	
FOLFIRINOX, n (%)	0 (0)	4 (3.3)	
5-FU, n (%)	0 (0)	2 (1.7)	
Gemcitabine + Afatinib, n (%)	1 (10.0)	12 (10.0)	
Capecitabin + Erlotinib, n (%)	0 (0)	1 (0.8)	
Ibrutinib/Placebo + nab-Paclitaxel + Gemcitabine, n (%)	1 (10.0)	8 (6.7)	
NAPOLI, n (%)	0 (0)	1 (0.8)	
Capecitabine, n (%)	1 (10.0)	2 (1.7)	
FOLFOX, n (%)	0 (0)	1 (0.8)	
Gemcitabine + nab-Paclitaxel + Afatinib, n (%)	0 (0)	2 (1.7)	
Bilirubin level [mg/dL] BL, median (minimum; maximum)	0.3 (0.2;1.7)	0.6 (0.2;14.2)	0.157
LDH [U/L] BL, median (minimum; maximum)	209 (179;393)	230 (13;1870)	0.439
CRP [mg/L] BL, median (minimum; maximum)	28 (4;65)	11 (1;348)	0.498
CA 19–9 [U/mL] BL, median (minimum; maximum)	35 (2;885)	978 (1;938,670)	0.003
CEA [μg/L] BL, median (minimum; maximum)	4 (2;80)	11 (0;13,006)	0.140

### Laboratory results and tumor markers

Baseline, highest and latest value of c-reactive protein (CRP), bilirubin and lactate dehydrogenase (LDH), as well as the tumor markers CA 19–9 and CEA, were examined (summarized data shown in Table 1). There was a significant difference in the baseline values of CA 19–9 (*p* = 0.003) between the OMD group and the remaining patients, which is explained by the threshold of 1000 U/mL we chose to define the OMD group. Mann-Whitney U Test for independent samples did not reveal a significant difference between bilirubin baseline, LDH baseline, CRP baseline and CEA baseline between the OMD and non-OMD groups (see Table 1).

### Survival analysis

**Fig. 3:**
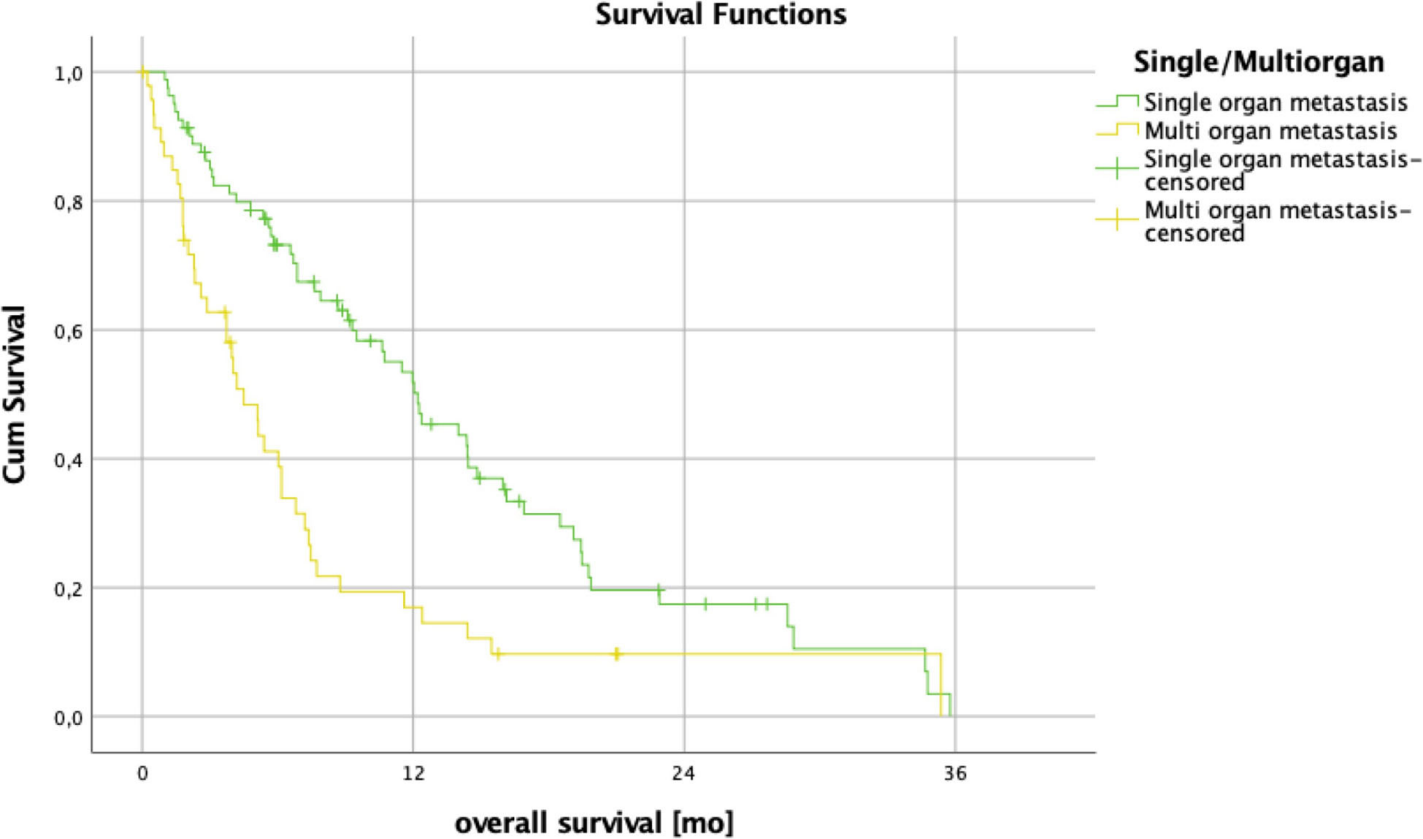
Survival analysis of patients with metastases confined to one organ upon diagnosis (single organ metastasis, SOG) vs patients with at least two metastastic organ manifestations. Overall survival was significantly longer in the single organ metastasis group with a median overall survival of 12.2 months vs 4.5 months (95% CI 5.7–9.8; *p* = < 0.001)

**Fig. 4:**
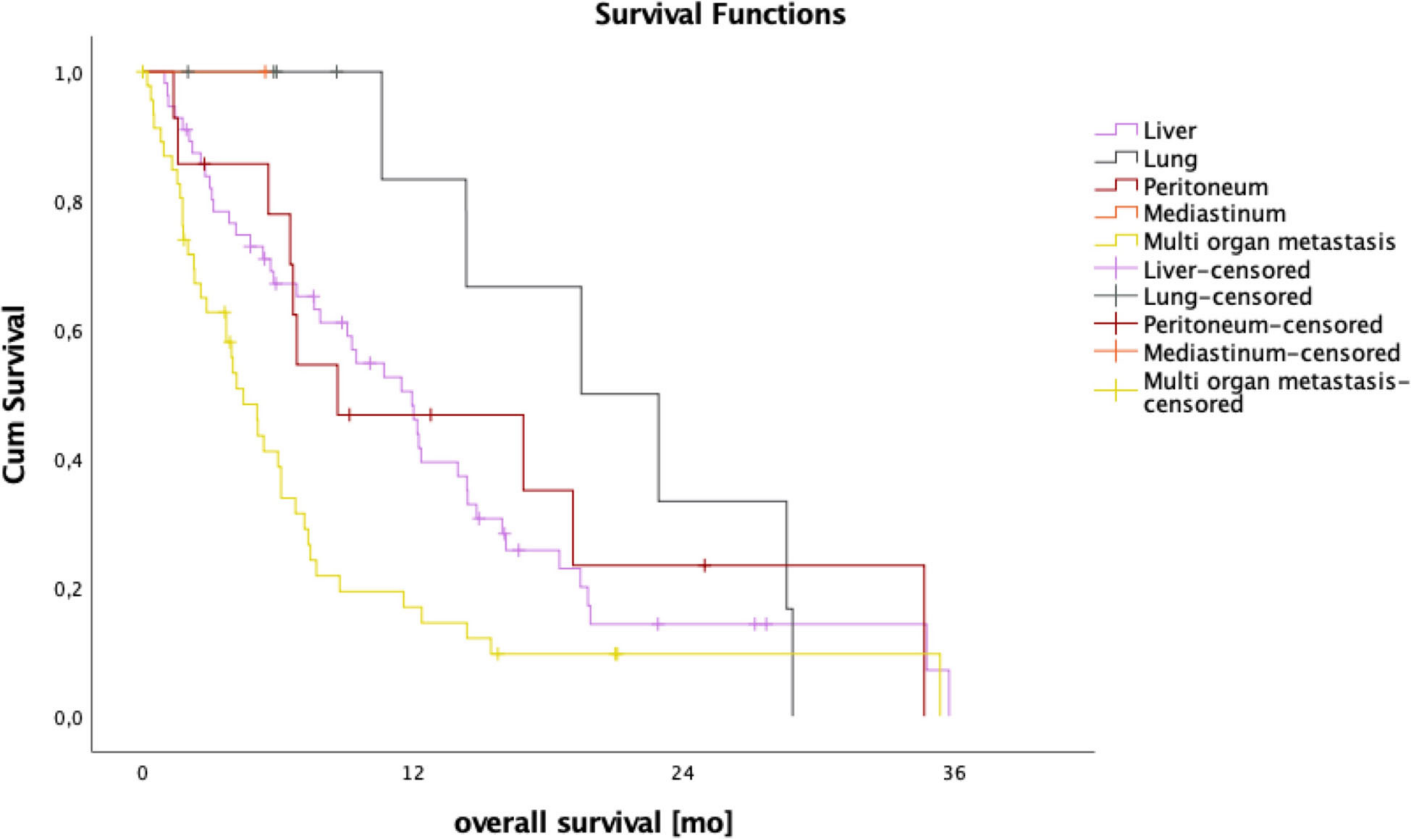
Kaplan-Meier analysis of single organ vs multi-organ metastasized patients according to organ manifestation in single organ group. Median overall survival of single organ liver, peritoneum and lung was 12.0, 8.7, and 19.5 months, respectively

**Fig. 5:**
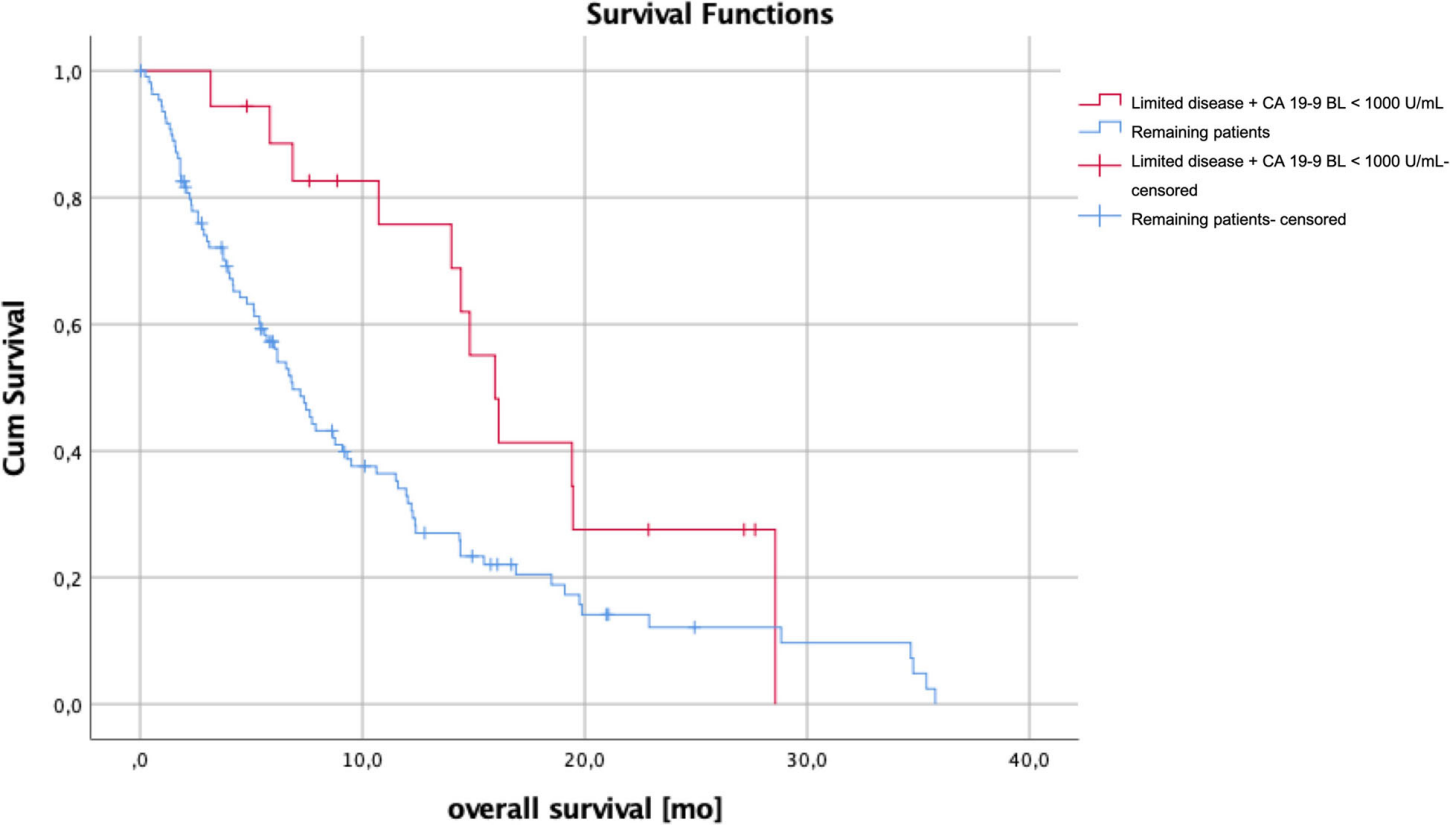
The survival analysis of patients with limited disease + CA 19–9 BL < 1000 U/mL revealed a significantly better overall survival of 16.0 months compared to the rest of the collective (6.9 months; 95% CI 5.7–9.8; *p* = 0.021)

**Fig. 6:**
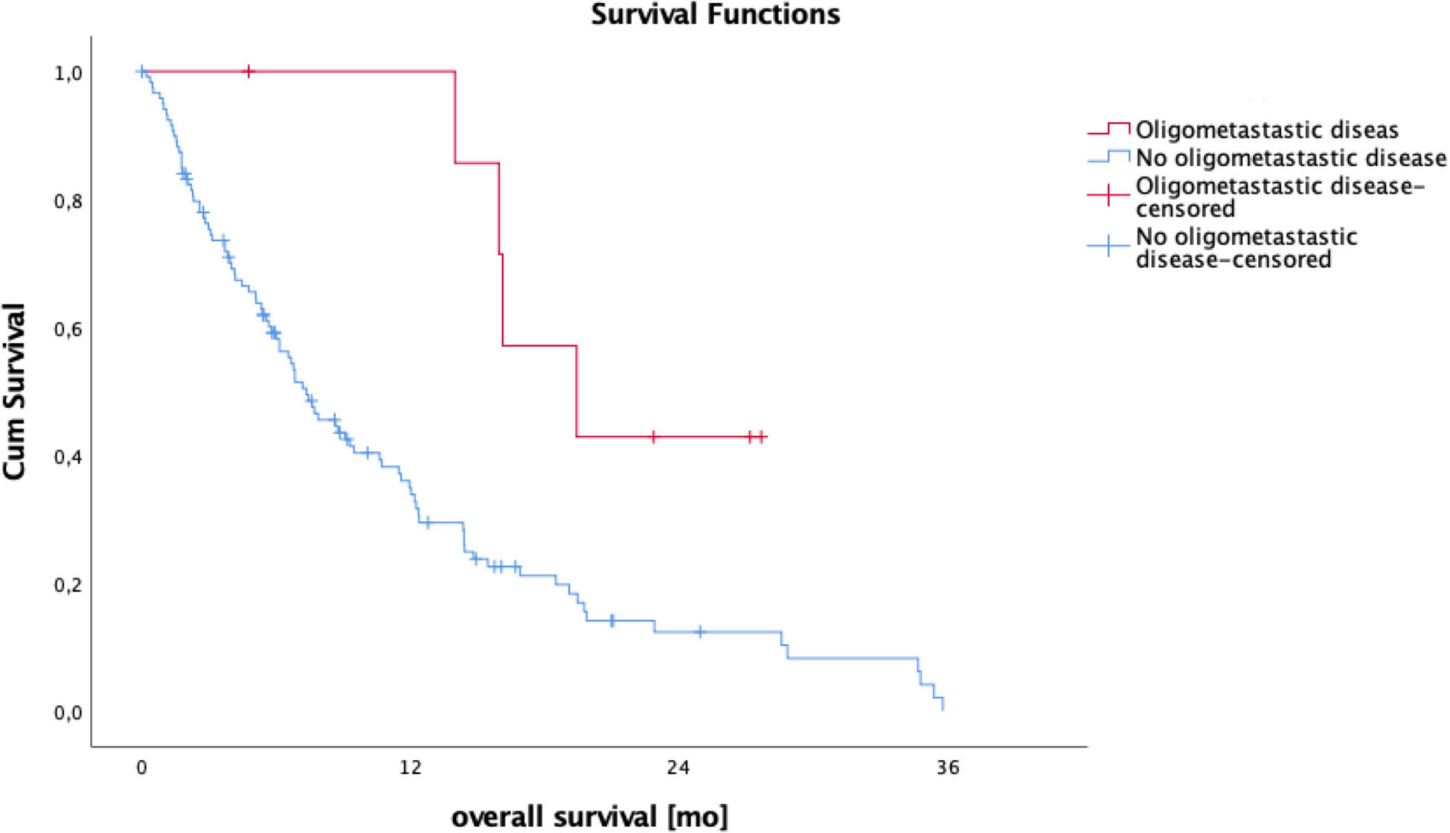
Patients fulfilling criteria for oligometastatic disease, as shown in Table 1, show a significantly longer median overall survival compared to the remaining patients (19.4 vs 7.2 months; 95% CI 5.7–9.8; *p* = 0.009)

Patients with a SOG pattern independent of the metastatic site showed a significantly longer median overall survival of 12.2 months compared to 4.5 months for MOG patients (95% CI 5.7–9.8; *p* = < 0.001; Fig. 3). Compared to the remaining collective, median overall patient survival of liver-only (12.0 vs 6.7 months; 95% CI 5.7–9.8; *p* = 0.148), peritoneum-only (8.7 vs 7.7 months; 95% CI 5.7–9.8; *p* = 0.439) and lung-only (19.5 vs 7.2 months; 95% CI 5.7–9.75; *p* = 0.072) involvement showed no statistically significant difference (Fig. 4). As we thought to identify patients with anatomically *limited disease*, we split single-organ liver or lung-metastasized patients into those having up to 4 and 5 or more metastases. The survival analysis did not reveal any significant difference between these groups (14.0 months vs 7.6 months, *p* = 0.164), indicating that *limited disease* and OMD are distinct entities. To approach OMD using a biological marker, we further categorized the *limited disease* group with up to 4 metastases into those with a baseline CA 19–9 below 1000 U/mL and above 1000 U/ml (Fig. 1). Here, survival analysis revealed a significantly longer median overall survival of 16.0 months vs 6.9 months in favor of the low CA 19–9 group (95% CI 5.7–9.8; *p* = 0.021, Fig. 5). Finally, to define the OMD, we added stable disease or response to first-line chemotherapy as another criterion representing greater benign tumor biology. Both lung-only patients had stable disease or response, whereas of the 16 patients with *limited disease* to the liver and CA 19–9 below 1000 U/mL, 8 (50%) showed response or stable disease. Referring to our collective, 10 (7.8%) patients fulfilled all criteria we used to define the OMD in PDAC. These OMD patients showed a superior median overall survival compared to the remaining 118 patients (non-OMD) of 19.4 vs 7.2 months (95% CI 5.7–9.8; *p* = 0.009, Fig. 6).

## Discussion

In our study, we analyzed patients with histologically proven pancreatic ductal adenocarcinoma and distant metastasis upon diagnosis. We hypothesized that the OMD in pancreatic cancer could be defined by the combination of anatomically limited disease with clinical parameters reflecting a better prognosis and a potentially more benign tumor biology.

Our aim was to gain further insight into PDAC M1 patients and to identify OMD patients with a beneficial prognosis. To our knowledge, no data is available describing the correlation between the clinical course and the metastatic pattern upon diagnosis of PDAC. All patients with a clinical stage IV PDAC are referred to palliative treatment following clinical guidelines [5, 6]. We could show that patients with a single organ metastasis (SOG) had a significantly longer overall survival than patients with multi-organ metastasis (MOG) upon diagnosis (12.2 months vs 4.5 months; 95% CI 5.7–9.8; *p* = < 0.001, Fig. 3). Also, within the SOG group, we observed differences in survival, which we identified using specific patients’ characteristics.

### Palliative chemotherapy

Patient data was retrospectively obtained from 2009 to 2018. As the studies adjusting the palliative chemotherapy regimen by von Hoff (Gemcitabine and nano-albumin-bound Paclitaxel) [7] and Conroy (FOLFIRINOX) [4] were both published in 2011, chemotherapy regimens of the included patients differ. Modern combination therapies with Gemcitabine and nano-albumin-bound Paclitaxel was administered in 21 of the 128 cases, but the more aggressive FOLFIRINOX regimen was administered in only 4 cases as a first line therapy. Proportions of chemotherapies between the OMD and the non-OMD group are shown in Table 1. The higher proportion of combination therapy in the OMD group could pose a bias with respect to the improved overall survival. However, the strength of the current data set lies in the presentation of the clinical reality with respect to chemotherapy regimens administered, since only a smaller portion of the patients were included in clinical trials.

### Focus on metastasized pancreatic adenocarcinoma confined to the liver or the lung

Hepatic spread was the predominant site of metastasis in our collective, both in the SOG (56 of 81, 69%) and in the MOG (43 of 47, 91%), underlining the clinical importance of hepatic metastases in stage IV PDAC (Fig. 1). This is also reflected by the fact that most studies that have analyzed a surgical approach to PDAC M1 patients focused on synchronous hepatic metastases treatment when there was a limited number of metastases [11, 12, 16–20]. In this light, many studies used the term *oligometastasis* synonymously with *limited disease* of the liver or lung as a justification for extended metastasis resection.

A concept of oligometastatic disease has been substantiated in recent years by various studies of gastrointestinal tumor diseases [21]. In colorectal carcinoma or renal cell carcinoma there is a survival advantage after resection of hepatic metastases [22, 23]. However, the concept of oligometastasis in PDAC is not taken into account in the current treatment guidelines.

Therefore, data on overall survival in PDAC M1 patients with hepatic or lung metastasis undergoing surgical resection are limited with a lack of prospective studies and mostly small sample sizes. A retrospective analysis of Hackert et al. [12] showed a median survival of 12.3 months in a group of 85 patients after radical surgery for PDAC with liver metastases. Tachezy and Gebauer [11] described a significantly longer median overall survival of 14.5 months vs 7.5 months in patients undergoing hepatic resection vs palliative bypass surgery in a cohort of 138 patients with liver metastasis. In a recent case-control study with a small sample size of a highly selected cohort, Kandel and colleagues [24] reported an overall survival of 32.4 months in the M1 surgery group vs 11.7 months in the M1 no-surgery group. They considered oligometastatic disease when there were < 2 metastatic tumors in liver or lung that were below 4 cm in size and patients had received FOLFIRINOX or Gemcitabine/nab-Paclitaxel upfront. Another recent publication by Frigerio and colleagues [25] reported an astoundingly high overall survival of 56 months in an, again, highly selected group of PDAC M1 patients with liver metastasis upon diagnosis and surgical resection of the primary tumor. In this study, patients were referred to resection only when restaging after neoadjuvant chemotherapy showed a disappearance of liver metastasis [25]. In our collective of patients treated with palliative chemotherapy, median overall survival of the 56 patients with single organ liver metastasis was not statistically significantly longer compared to the remaining patients (Fig. 5). The majority (*n* = 34) of those liver-only patients had disseminated metastatic spread in both liver lobes.

Regarding patients with single organ metastases in the lung, data is scarce because isolated pulmonary metastases are rare in stage IV PDAC. In the 81 SOG patients, only 10 (12%) had pulmonary metastases and the majority (*n* = 8, 10%) showed disseminated spread in the lung. The difference in median overall survival (lung only, 19.5 vs remaining patient collective, 7.2 months; 95% CI 5.7–9.8, *p* = 0.072) was not statistically significant, albeit it was more than twice as long. Kandel and colleagues [24] treated 2 patients with isolated metastases to the lung with radiofrequency ablation, with one of the patients showing the best overall survival in the whole group with 3.7 years. In a 2011 retrospective study by Arnaoutakis and colleagues [10, 26]. Nine patients with metachronous pulmonary metastasis underwent surgical resection of lung metastases and showed a survival benefit of 52 vs 22 months (*p* = 0.04) over patients with isolated pulmonary metastases that did not undergo resection.

### *Limited disease* as a first step to approach the OMD concept

In the prospect of a possible surgical R0 resection of the metastases, we limited the number of metastases in the liver and the lung to a maximum of 4 to define *limited disease*. This is in accordance with the number of liver metastases that had been resected in the retrospective works of Hackert et al. and Tachezy et al., even though no strict definition had been applied before [11, 12]. Also, the same number of 4 metastases had been identified by Fahy et al. as a factor associated with better overall survival in colon cancer with synchronous hepatic metastases [27]. Even though transferring concepts to PDAC patients has to be done with caution, we believe that the concept of oligometastasis could apply to all solid organ malignancies [21]. The cut-off of 4 metastases was also chosen with regard to a limited extension of hepatic resection in patients undergoing major pancreatic surgery. Here, we consider major hepatectomy (right hepatectomy or more) at the time of pancreatic tumor resection as not feasible in most cases. Preferably, atypical resection or ablative procedures should suffice in hepatic resection in order to limit morbidity. In our collective, 21 of 57 patients (37%) with single organ hepatic metastases had a maximum of 4 metastases upon diagnosis (Fig. 1). Survival analysis did not reveal a significant difference in overall survival between both groups (maximum of 4 metastases vs 5 and more metastases; 14.0 vs 7.6; *p* = 0.164).

### From anatomically *limited disease* to oligometastatic disease

These findings support the idea that anatomically limited disease alone is not sufficient to define oligometastatic disease as a condition with favorable prognosis that might qualify for surgery. In accordance with this assumption, the recent American Society of Clinical Oncology guidelines add biological criteria to the anatomical definition of borderline resectable PDAC (BR-PDAC) [28]. Isaji et al. define biological BR-PDAC as the presence of regional lymph node metastasis, radiologically or biopsy proven, and CA 19–9 levels of more than 500 U/mL [29].

Many studies have examined the importance of tumor markers in pancreatic cancer and CA 19–9 has been shown to be of special value in characterizing the clinical course of pancreatic cancer. CA 19–9 response to neoadjuvant therapy can help to predict the outcome in PDAC, and CA 19–9 was described as a marker of resectability and survival in PDAC [30, 31]. Also, CA 19–9 may show response to chemotherapy in PDAC M1 patients before the response is evident in radiological imaging evaluated by RECIST criteria [32]. Hartwig et al. described that a CA 19–9 level of more than 1000 U/mL reduces the number of R0 resections significantly to 15% [33]. We applied this criterium to our cohort of patients and identified eighteen of 21 patients with a maximum of 4 metastases in the liver or the lungs had a CA 19–9 below 1000 U/mL upon diagnosis (limited disease + CA 19–9, see (Fig. 1). The survival analysis revealed a significantly better overall survival of 16.0 months in this group compared to the rest of the collective (7.7 months; *p* = 0.021, see (Fig. 5).

Still, one of the main considerations in primary tumor and metastasis resection in PDAC should be to enable patient selection with a less aggressive tumor biology reflected as well by response to preoperative chemotherapy. We included this in our definition of oligometastatic disease to further distinguish the OMD group from *limited disease*.

Recently published and ongoing trials clearly hint at a benefit for neoadjuvant therapy in non-metastasized PDAC [34–36]. A national cancer database query identified 15,237 patients with resectable PDAC; 13,000 underwent upfront surgery and 2000 underwent neoadjuvant treatment. A propensity score-matched analysis revealed a significant median overall survival benefit for the patients that had received neoadjuvant therapy (26 vs 21 months) [34]. Mirkin et al. supported those findings by an analysis of a similar dataset showing a benefit for stage III patients (pT4) [37]. Even though the previously mentioned studies do not cover PDAC M1 patients, we would consider upfront systemic therapy with subsequent reevaluation a necessity in order to prevent unjustified surgical treatment of PDAC M1 patients. This “test of time” helps select patients with a better tumor biology. Of the 18 patients with </=4 hepatic or lung metastases and a CA 19–9 below 1000 U/mL upon diagnosis, 10 patients (8 with liver metastases and 2 with lung metastases) showed stable disease or response in the radiological evaluation in the first or second restaging after systemic treatment (Table 2). Again, survival analysis of this cohort revealed a significant survival benefit of 19.4 months compared to the whole cohort with 7.2 months (*p* = 0.009, see (Fig. 6). Thus, we believe that OMD can be described using our definition. In order to put the OMD group’s overall survival into the context of surgical PDAC treatment of M0 patients, we analyzed median overall survival at our institution since mid 2016, which was 22.3 months (95%CI 17.9-not yet reached), respectively (Fig. 7). Expectedly, the resected patient’s median OS was better compared to OMD. Still, the difference of only approximately 3 months underlines that according to our definition of OMD, a patient cohort can be identified that shows a median OS which is much closer to PDAC M0 resected patients, than to the remaining M1 patients not matching our criteria (7.2 months). Another group of patients are those with non-metastasized unresectable PDAC. For those, that remain unresectable even after neoadjuvant treatment an overall survival of 12–18 months is mentioned in the literature [38]. Again, OMD patients in our cohort could reach an even better survival than non-metastasized patients. Could this be verified in a greater cohort of PDAC M1 patients, it would mean that resectable patients with oligometastatic disease may have an at least equal and at times even better OS than patients with localized disease.

**Table 2:** Overview of patients who, according to our criteria, are considered to have OMD

#	Study participation	Overall survival [mo]	Primary tumor: Max. diameter [cm]	Primary tumor: Location	M: Liver count	Liver max metastasis [cm]	M: Liver location	M: Lung count	M: Lung max. Diameter [cm]	Chemo: First line	Bilirubin level [mg/dL] BL	LDH [U/L] BL	CRP [mg/L] BL	CA 19–9 [U/mL] BL	CEA [μg/L] BL
1	No	16.1	4.1	Tail	4	4.1	Right lobe	–		Gemcitabine + nab-Paclitacxel	0.3	208	45.9	6	2.8
2	ACCEPT	16.0	5.0	Tail	3	6.3	Both lobes	–		Gemcitabine + Afatinib	0.3	393	63.1	22	80.2
3	No	22.9	n.a.	Tail	2	4.4	Both lobes	–		Gemcitabine + Erlotinib	1.2	209	64.9	33	1.7
4	No	27.7	2.4	Head	4	1.1	Both lobes	–		Gemcitabine + nab-Paclitacxel	1.7	246	32.5	885	7.7
5	RESOLVE (PCYC-1137-CA)	14.0	2.9	Tail	4	1.4	Right lobe	–		Ibrutinib/Placebo + nab-Paclitacxel + Gemcitabine	0.7	180	6.0	229	1.6
6	No	19.4	4.6	Body	2	0.9	Left lobe	–		Gemcitabine + Erlotinib	0.2	198	4.3	154	3.8
7	No	4.8	3.6	Head	3	0.9	Both lobes	–		Gemcitabine	0.3	179	23.1	2	3.2
8	No	27.2	4.2	Body	4	1.6	Right lobe	–		Gemcitabine	0.3	230	5.0	36	7.6
9	No	28.6	9.0	Body	–	–	–	4	4.5	Capecitabine	0.2	276	9.2	790	1.8
10	No	19.5	4.8	Body	–	–	–	2	0.5	Gemcitabine	0.5	256	3.0	148	2.6

**Fig. 7:**
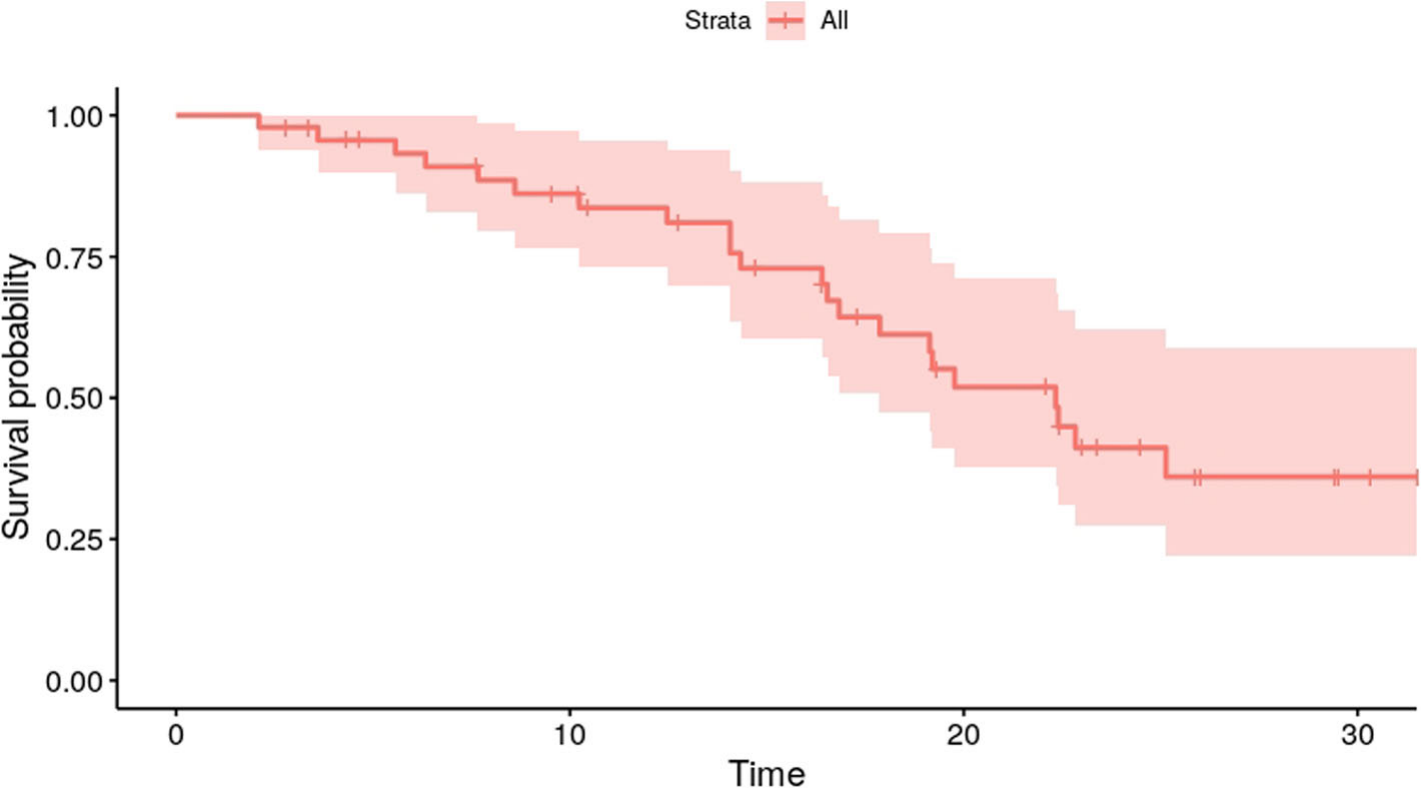
Overall survival of patients with non-metastasized PDAC (M0) at our institution since mid 2016 is 22.3 months (*n* = 47)

### Patient factors in metastasis surgery in PDAC

The OMD patients might receive a different type of treatment—including surgery—in the future. One of the limitations of the retrospective design is that these patients had not been clinically evaluated with respect to surgery. Pancreatic surgery for cancer is associated with a mortality of up to 10%, even in high-volume centers [39, 40]. Procedures for the resection of PDAC, especially pancreatic head resection performed as a Traverso or Whipple procedure, are major surgeries with a high morbidity. Therefore, individual patients’ factors, foremost comorbidities, must be taken into account in the treatment recommendation. Even though we would not encourage synchronous major hepatic resection in PDAC M1 patients, minor resection and ablative procedures will likely go along with an increased operation time and possibly blood loss. The same will apply to radiofrequency ablation, cyber knife and lung resections for pulmonary metastases. We therefore suggest the inclusion of the Eastern Cooperative Oncology Group performance status (ECOG) to help determine eligibility for surgery in our criteria for oligometastatic disease in PDAC M1.

## Conclusions

We defined oligometastatic disease in pancreatic cancer by a combination of morphological criteria of the metastases (*limited disease*), prognostic markers (CA 19–9) and the individual patient’s tumor biology judged by response to systemic therapy (Table 3). Application of these criteria revealed a subgroup of 10 (7.8%) PDAC M1 patients with a significantly better survival. In our opinion, the broad spectrum of clinical courses observed in M1 patients requires individualized clinical pathways for these patients and underlines that PDAC M1 patients are a very heterogeneous group. Drawing a conclusion concerning therapeutic consequences is too early at this point. Clinical studies will need to be conducted to validate our criteria. Nonetheless, evidence is accumulating that in other aggressive cancer entities such as gastric cancer, resection of the primary tumor in combination with hepatic metastases resection is beneficial for the patient [41]. Our rationale to extend the therapy of PDAC M1 patients to surgical resection rests on the assumption that the already comparatively very good median overall survival of the OMD group can be further improved. Our OMD criteria reflecting the clinical course in combination with anatomical and biological factors are a possible instrument to identify patients whose favorable tumor biology justifies the maximum multimodal therapy, including surgery. We therefore believe that “M1 is not M1” and in the context of multimodal treatment, patients identified by our OMD criteria could potentially benefit from surgery.

**Table 3:** Summary of all criteria as proposed by us for the definition of the oligometastatic disease in pancreatic cancer

Criteria for Oligometastatic Disease in Pancreatic Ductal Adenocarcinoma		
	Inclusion	Exclusion
ECOG	0/1	>/= 2
Liver/lung only (+LN hepatoduodenal lig.)	Yes	No
Number of hepatic or pulmonary lesions	</= 4	> 4
Ascites	No	Yes
Liver cirrhosis	No	Yes
Lung emphysema	No	Yes
CA 19–9 (U/mL)^a^	< 1000	> 1000
Surgery for metastasis^b^	Atypical resection (+/−Ablation)	Non-resectable/major hepatic/lung surgery necessary
Primary tumor upon diagnosis^b^	Resectable, borderline resectable, non-resectable^c^	
Chemotherapy response	Response or stable disease	Progressive disease

^a^Hartwig et al. 2013 Ann Surg Oncol (2013) 20:2188–2196; DOI 10.1245/s10434-012-2809-1: R0 Resection rate CA 19-9 > 1000U/ml as low as 15%

^b^Resectability of primary tumor and expected extension of hepatic resection to be evaluated by experienced HPB surgeon together with a radiologist

^c^As defined in: Isaji, S. et al. International consensus on definition and criteria of borderline resectable pancreatic ductal adenocarcinoma 2017. *Pancreatology* 18, (2018)

## Data Availability

The datasets used and/or analysed during the current study are available from the corresponding author on reasonable request.

## Abbreviations

5-FU: Fluorouracil
BR- PDAC: Borderline resectable pancreatic ductal adenocarcinoma
CA 19–9: Carbohydrate antigen
CEA: Carcinoembryonic antigen
CRP: C-reactive protein
CT: Computed tomography
ECOG: Eastern Cooperative Oncology Group performance status
FOLFIRINOX: Fluorouracil, Irinotecan, Oxaliplatin
LDH: Lactate dehydrogenase
MOG: Multi organ metastasis group
MRI: Magnetic resonance imaging
Nab-Paclitaxe: Nano-albumin-bound Paclitaxel
NAPOLI: Liposomal Irinotecan (nal-IRI) plus 5-fluorouracil and leucovorin (5-FU/LV)
OMD: Oligometastatic disease
OS: Overall survival
PDAC M1 HEP: Only hepatic metastasized pancreatic ductal adenocarcinoma
PDAC M1 PER: Only peritoneally metastasized pancreatic ductal adenocarcinoma
PDAC M1 PULM: Only pulmonary metastasized pancreatic ductal adenocarcinoma
PDAC M1: Metastasized pancreatic ductal adenocarcinoma
PDAC: Pancreatic ductal adenocarcinoma
RECIST: Response Evaluation Criteria In Solid Tumors
RESOLVE (PCYC-1137-CA): Study of Ibrutinib vs Placebo, in Combination With Nab-paclitaxel and Gemcitabine, in the First Line Treatment of Patients With Metastatic Pancreatic Adenocarcinoma
SOG: Single organ metastasis group
